# Quality of life in lung cancer survivors treated with tyrosine-kinase inhibitors (TKI): results from the multi-centre cross-sectional German study LARIS

**DOI:** 10.1007/s00432-022-03975-6

**Published:** 2022-05-24

**Authors:** Nicola Riccetti, Maria Blettner, Katherine Taylor, Beatrice Wehler, Bernhard Gohrbandt, Ursula Nestle, Robert Bals, Marcus Stockinger, Thomas Wehler, Susanne Singer, Martin Eichler

**Affiliations:** 1 University Medical Center, Johannes Gutenberg University, Langenbeckstraße 1, 55131 Mainz, Germany; 2 University Cancer Center Mainz, Langenbeckstraße 1, 55131 Mainz, Germany; 3 Universitätsklinikum Gießen Marburg GmbH Standort Gießen, Medizinische Klinik IV/V, Klinikstr. 33, 35392 Gießen, Germany; 4 Katholisches Klinikum Mainz, An der Goldgrube 11, 55131 Mainz, Germany; 5 University Medical Center Freiburg, Albert-Ludwig-University, Hugstetter Str. 55, 79106 Freiburg im Breisgau, Germany; 6 Boehringer-Ingelheim, Binger Str. 173, 55216 Ingelheim am Rhein, Germany; 7 Department of Internal Medicine V, Saarland University Medical Center, Kirrberger Str. 100, 66421 Homburg, Germany; 8 University Hospital Carl Gustav Carus, Fetscherstraße 74, 01307 Dresden, Germany; 9 Institute of Medical Biostatistics, Epidemiology, and Informatics (IMBEI), University Medical Center of the Johannes Gutenberg University, Obere Zahlbacher Straße 69, 55131 Mainz, Germany

**Keywords:** Quality of life, Symptom burden, Lung cancer, RTK genetic alterations, Tyrosine-kinase inhibitors

## Abstract

**Purpose:**

We aimed at exploring the quality of life (QOL) of lung cancer survivors with proven tyrosine-kinase receptor (RTK) genetic alterations and targeted tyrosine-kinase inhibitors (TKI) therapy, compared to lung cancer survivors with no-RTK alterations and no-TKI therapy.

**Methods:**

Data were collected in a cross-sectional multi-centre study. Primary lung cancer survivors were asked about their socio-demographic and clinical information, QOL, symptom burden, and distress. QOL and symptom burden were assessed using the European Organization for Research and Treatment of Cancer Quality of Life Questionnaire Core 30 (EORTC QLQ-C30), and distress with the Patient Health Questionnaire-4 (PHQ-4). Demographic and clinical characteristics were reported in absolute and relative frequencies, QOL, and symptom burden using mean scores. Differences in mean scores with relative 95% confidence intervals were used for comparison.

**Results:**

Three groups of survivors were defined: group A with proven RTK alterations, TKI therapy at any time during therapy, and stage IV lung cancer at diagnosis (*n* = 49); group B: non-TKI therapy and stage IV lung cancer (*n* = 121); group C: non-TKI therapy and stage I–III lung cancer (*n* = 495). Survivors in group A reported lower QOL (mean score difference = -11.7 vs. group B) and symptom burden for dyspnoea (difference = -11.5 vs. group C), and higher symptom burden for appetite loss (difference =  + 11.4 vs. group C), diarrhoea and rash (differences =  + 25.6, + 19.6 and + 13.2, + 13.0, respectively, vs. both groups).

**Conclusions:**

Our results suggest that the specific side effects of TKI therapy can impair QOL among lung cancer survivors. Therefore, specific focus towards the optimal management of these side effects should be considered.

## Introduction

Besides its high incidence and mortality, lung cancer presents a considerable symptom burden and impact on the quality of life (QOL) of the patients and survivors. This symptom burden manifests both physically (e.g. fatigue, loss of appetite, dyspnoea, cough, and shortness of breath) and psychologically (e.g. anxiety and depression) (Linden et al. 2012; Brintzenhofe-Szoc et al. 2009; Eichler et al. 2018; Akin et al. 2010). For these reasons, therapeutic approaches to lung cancer are evaluated not only for their bio-medical outcomes, but also for their impact on the QOL of the patients (Arraras et al. 2016; Braun et al. 2011; Iyer et al. 2013).

Tyrosine-kinase inhibitors (TKI) are a class of drugs that can be used in patients with tyrosine-kinase receptor (RTK) genetic alterations such as epidermal growth factor receptor (EGFR) mutations, anaplastic lymphoma kinase (ALK) gene rearrangements, or proto-oncogene receptor tyrosine-kinase (ROS1) gene rearrangements (Paz-Ares et al. 2010; Barlesi et al. 2016; Kashima et al. 2019). TKIs target these alterations, inhibiting the activation of the RTK.

TKIs were seen to improve the response rate and progression-free survival, as well as the QOL and symptom burden of cancer patients with RTK alterations and advanced disease, compared to patients treated with conventional chemotherapy (Batson et al. 2017; Yang et al. 2013; Wu et al. 2014). More patients treated with *Gefitinib* reported an improvement in their QOL over time as well as longer time to deterioration for pain and shortness of breath, than the ones treated with carboplatin and paclitaxel (*p* = 0.042) and chemotherapy [hazard ratio (HR) 0.34; 95% confidence interval (CI) 0.23, 0.50], respectively (Oizumi et al. 2012a). Lung cancer patients with exon 19 in-frame deletions treated with *Afatinib* reported longer time to deterioration of their QOL, compared to patients receiving chemotherapy (HR 0.53; 95% CI 0.35, 0.82) (Wu et al. 2018). More patients with exon 19 in-frame deletions and exon 21 L858R substitutions treated with *Afatinib* reported an improvement in their QOL, than patients receiving chemotherapy (63% vs 34%; *p* < 0.001; and 61% vs 34%; *p* = 0.007, respectively) (Wu et al. 2018).

In addition, patients treated with TKIs reported lower symptom burden for dyspnoea and cough than patients receiving chemotherapy (Wu et al. 2018). When compared to patients treated with chemotherapy, more patients treated with *Afatinib* reported relived dyspnoea (64% vs 50%; *p* = 0.10) and improvement in shortness of breath (57% vs 36%; *p* < 0.001). Furthermore, these patients reported a significantly longer time until deterioration for cough (HR 0.68; 95% CI 0.50, 0.93) (Yang et al. 2013).

These improvements in QOL or symptom burden in patients treated with TKIs are often partial and/or temporary, with the development of resistance to TKIs often occurring already 6–12 months after the beginning of the therapy (Rotow and Bivona 2017; Sibilia et al. 2007). Moreover, TKIs might generate cutaneous and gastro-enteric side effects, due to the expression of EGFR in the skin and gastro-enteric epithelial cells (Hirsh 2011). Stomatitis, mucositis, rash, dry skin, and paronychia are the most reported cutaneous side effects of TKIs, while diarrhoea is the most commonly reported gastro-enteric one (Califano et al. 2015). Among lung cancer patients treated with *Osimertinib,* 44% and 42% reported diarrhoea and rash, respectively, followed from dry skin (29%), paronychia (27%), decreased appetite (18%), and stomatitis (16%) (Yi et al. 2019). Patients treated with *Afatinib* reported a shorter time to deterioration for diarrhoea and sore mouth, compared to patients treated with chemotherapy (Wu et al. 2018). Although often present only in a mild form, these side effects can have a detrimental effect on the QOL of the patients and can cause modifications in the type and posology of the therapy (Califano et al. 2015).

All the aforementioned papers on the beneficial effects of TKIs on the QOL and symptom burden of the patients are based on data collected in clinical trials and on comparable populations of patients with and without RTK genetic alterations and targeted TKI therapy. Conversely, few information is available on the late effects of the therapy with TKIs and the QOL and symptom burden of lung cancer patients and survivors with targeted-TKI therapy, in a real-world population.

Therefore, the aim of this analysis was to explore the QOL of lung cancer survivors with proven RTK genetic alterations and targeted TKI therapy at any time during the course of their treatment, compared to the QOL of lung cancer survivors with no-RTK genetic alteration and no-TKI therapy.

## Patients and Methods

### Data collection

Data collection took place between 2015 and 2016 in the cross-sectional, multicentre, German study LARIS (Quality of Life and Psychosocial Rehabilitation in Lung Cancer Survivors). In this study, primary lung cancer survivors and patients who had survived at least one year after the diagnosis were enrolled. Further inclusion criteria were: (1) at least 18 years of age, (2) lung cancer-related admission to the hospital between 2004 and 2014, and (3) being mentally and verbally able to take part in a telephone interview in German (Eichler et al. 2018; Hechtner et al. 2019; Rashid 2021).

Participant hospitals were the University Hospitals in Mainz, Frankfurt, Leipzig, Freiburg, and Homburg, and the Catholic Hospital in Mainz. The tumour registries of these hospitals were used to identify potential participants, which were contacted directly by each institution. After returning the informed consent, participants completed a questionnaire and took part in an interview. The interviews provided socio-demographic and other personal information (e.g. living situation, psychosocial care), the questionnaires provided information on the QOL and symptom burden, while treatment and tumour-specific data were collected from the patients’ medical records. Non-responders were contacted with up to two reminder letters including all invitation documents and the questionnaires (Eichler et al. 2018; Hechtner et al. 2019).

The study protocol was approved by the Ethics Committee of the Medical Chamber Rhineland Palatinate before the beginning of the interview phase (n. 837.376.14), and the study was conducted in accordance with the Declaration of Helsinki.

### Instruments

QOL was assessed using the European Organisation for Research and Treatment of Cancer (EORTC) Quality of Life Questionnaire Core 30 (QLQ-C30) (Aaronson et al. 1993). This questionnaire is a self-reported measure which consists of: (1) a two-item global quality-of-life scale, (2) five multi-item functional scales (physical, role, cognitive, emotional, and social), and (3) nine symptom scales, three of which are multi-item symptom scales (fatigue, nausea and vomiting, and pain) and six are single-item symptom scales (dyspnoea, insomnia, appetite loss, constipation, diarrhoea, and financial impact) (Giesinger et al. 2016). Considering the frequency of cutaneous side effects among patients treated with TKI, a single-item symptom scale for rash from the EORTC library was included in the questionnaire.

Each scale in the questionnaire is rated by the participant on a 4-point Likert scale ranging from “not at all” (0) to “very much” (3). The global QOL scale presents a 7-point scale ranging from “very poor” (1) to “excellent” (7). Each raw score is then standardized to range between 0 and 100 (Fayers et al. 2001). Higher scores in the global QOL scale and functioning scales indicate better QOL, while high scores in the symptoms scales indicate higher symptom burden (Fayers et al. 2001).

Psychological distress was evaluated using the Patient Health Questionnaire-4 (PHQ-4) (Kroenke et al. 2009). The PHQ-4 comprises both core diagnostic criteria for depression and anxiety. In the PHQ-4, the items are rated on a 4-point Likert scale ranging from “not at all” (0) to “nearly every day” (3). A sum score for each scale of ≥ 3 is considered as cut-off for the presence of depression or anxiety.

### Statistical analysis

Participants were divided into three groups based on presence of genetic alterations, therapy with TKIs, and stage of cancer. The demographic characteristics of the participants, their medical information, the state of their disease, and their psychological distress were reported in their absolute and relative frequencies. Differences in proportions of psychological distress among the groups were calculated with the relative 95% CI (Rothman et al. 2008).

The results of the EORTC QLQ-C30 scales were reported using mean values and standard deviations. In this paper, we included only the results of specific EORTC QLQ-C30 scales (global quality-of-life, physical functioning, fatigue, nausea and vomiting, pain, appetite loss, diarrhoea, rash, and dyspnoea). However, to avoid giving an incomplete or biased picture of the QOL of the participants, the results of all the EORTC QLQ-C30 scales were provided in the supplementary material. Differences in mean scores among the groups with the relative 95% CI were calculated (Rothman et al. 2008). For the interpretation of these differences, previous works on the clinical relevance of the difference in mean scores of the EORTC QLQ-C30 scales were included (King 1997; Osoba et al. 1998; Cocks et al. 2011) (Table 1).

**Table 1:**
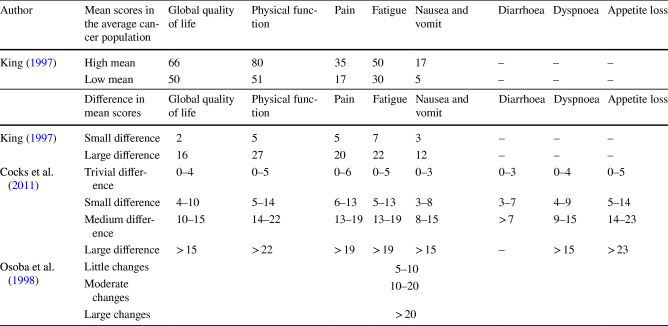
Summary of the clinically relevant mean scores and differences in mean scores for each scale from the European Organization for Research and Treatment of Cancer Quality of Life Questionnaire Core 30 (EORTC QLQ-C30) included in the analysis

For both the comparisons in terms of proportions of psychological distress and of mean scores, we considered testing exploratory two-sided hypotheses. In accordance with the explorative nature of this paper, no analysis of confounders was conducted.

## Results

### Patient selection

From the original 717 individuals (56% of the initially contacted *N* = 1287) who took part in the LARIS study, 52 were excluded from this analysis: 14 for not presenting with a confirmed RTK alteration, 23 for not having a confirmed cancer status, and 15 because information on the presence or absence of a TKI therapy was missing.

**Fig. 1:**
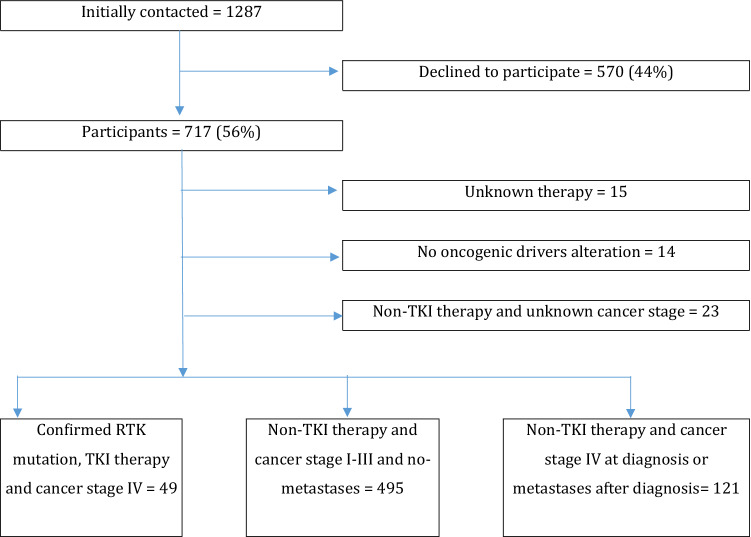
Study population chart

The remaining 665 individuals were included: 49 presented confirmed RTK genetic alterations, confirmed TKI therapy at any point in the therapy, and stage IV cancer at diagnosis (group A); 121 had no-RTK alterations, confirmed non-TKI therapy and cancer stage IV at diagnose or metastases after diagnosis (group B); and 495 had no-RTK alterations, confirmed non-TKI therapy, and cancer stage I–III at diagnosis and no metastases (group C) (Fig. 1).

### Demographic characteristics of the sample

The mean age at diagnosis was 58.4 years in group A, 60.1 years in group B, and 63.9 years in group C. The mean time since diagnosis was 3.7 years in group A, 4.1 years in group B, and 4.6 years in group C.

At the time of the data assessment, 23 survivors (19.0%) in group B and 68 (13.7%) in group C reported being active smokers. No participant from group A reported being an active smoker. *N* = 23 patients (46.9%) from group A, 83 (68.6%) from group B, and 360 (72.7%) from group C reported being former smokers.

### Clinical characteristics of the sample

Based on clinical data, 39 individuals (79.6%) from group A, 33 (27.3%) from group B, and 43 (8.7%) from group C were in treatment at time of the data assessment. For 4 (8.2%) in group A, 61 (50.4) in group B, and 393 (79.4%) in group C, the treatment was ended at the time of the data assessment.

*N* = 23 individuals (46.9%) in group A, 80 (66.1%) in group B, and 361 (72.9%) in group C presented with at least one comorbidity (Table 2).

**Table 2:** Demographic and clinical characteristics of the study sample

Covariates		Group A *N* = 49 (targeted-TKI treatment, Stage IV at diagnosis)	Group B *N* = 121 (no-TKI treatment, Stage IV at diagnosis or metastases)	Group C *N* = 495 (no TKI, Stage I–III at diagnosis, no metastases)
Age at diagnosis (mean/SD)		58.4/12.4	60.1/9.2	63.9/9.4
Time since diagnosis in years (mean/ SD)		3.7/2.5	4.1/3.3	4.6/ 2.8

		*N* (%)	*N* (%)	*N* (%)
Sex				
Male		20 (40.8)	62 (51.2)	320 (64.6)
Female		29 (59.2)	59 (48.8)	175 (35.4)
Age groups (age in years at interview)				
< 50		5 (10.2)	2 (1.7)	9 (1.8)
50–59		14 (28.6)	35 (28.9)	75 (15.2)
60–69		16 (32.7)	51 (42.1)	176 (35.6)
> 70		14 (28.6)	33 (27.3)	235 (47.5)
Smoking status (self-reported, and clinical data)				
Never		19 (38.8)	11 (9.1)	38 (7.7)
Former		23 (46.9)	83 (68.6)	360 (72.7)
Current		0	23 (19.0)	68 (13.7)
Unknown		7 (14.3)	4 (3.3)	29 (5.9)
Partner				
No		6 (12.2)	31 (25.6)	81 (16.4)
Yes		33 (67.3)	76 (62.8)	332 (67.1)
Missing		10 (20.4)	14 (11.6)	82 (16.6)
Social well-being				
Low		9 (18.4)	30 (24.8)	145 (29.3)
High		38 (77.6)	81 (66.9)	298 (60.2)
Missing		2 (4.1)	10 (8.3)	52 (10.5)
Employment				
Employed		10 (20.4)	10 (8.3)	50 (10.1)
Unemployed		0	8 (6.6)	8 (1.6)
Housewife/man		1 (2.0)	7 (5.8)	7 (1.4)
Disability pension		8 (16.3)	21 (17.4)	42 (8.5)
Retired		20 (40.8)	60 (49.6)	303 (61.2)
Not asked/unknown		10 (20.4)	15 (12.4)	85 (17.2)
Household income (in €)				
< 1000		2 (4.1)	15 (12.4)	36 (7.3)
1000–< 2000		8 (16.3)	40 (33.1)	152 (30.7)
2000–< 3000		8 (16.3)	27 (22.3)	107 (21.6)
3000–< 4000		9 (18.4)	9 (7.4)	45 (9.1)
> 4000		7 (14.3)	7 (5.8)	38 (7.7)
Declined to report		5 (10.2)	9 (7.4)	35 (7.1)
Not asked		10 (20.4)	14 (11.6)	82 (16.6)
Former or current occupation				
Blue collar worker		6 (12.2)	20 (16.5)	83 (16.8)
Civil servant		4 (8.2)	5 (4.1)	31 (6.3)
White collar worker		25 (51.0)	63 (52.1)	224 (46.9)
Self-employed		4 (8.2)	14 (11.6)	53 (10.7)
Missing		10 (20.4)	19 (15.7)	104 (21.0)
Education				
None to lower secondary education		13 (26.5)	56 (46.3)	246 (49.7)
Secondary school leaving certificate		12 (24.5)	26 (21.5)	86 (17.4)
(Professional) High school certificate		14 (28.6)	25 (20.7)	80 (16.2)
Missing		10 (20.4)	14 (11.6)	83 (16.8)
Current treatment (self-reported)				
In treatment		23 (46.9)	21 (17.4)	15 (3.0)
Not in treatment		16 (32.7)	86 (71.1)	399 (80.6)
Missing		10 (20.4)	14 (11.6)	81 (16.4)
Treatment status (clinical data)				
In treatment		39 (79.6)	33 (27.3)	43 (8.7)
Treatment ended		4 (8.2)	61 (50.4)	393 (79.4)
Unknown		5 (10.2)	16 (14.1)	36 (7.2)
Therapy planned		1 (2.0)	11 (9.1)	23 (4.6)
At least one comorbidity (cancer, diabetes, kidney, cardiovascular, respiratory), (self-reported + clinical data)				
No		21 (42.9)	40 (33.1)	123 (24.8)
Yes		23 (46.9)	80 (66.1)	361 (72.9)
Missing		5 (10.2)	1 (0.8)	11 (2.2)

### Genetic alterations and targeted treatment

*N* = 30 individuals in group A tested positive for EGFR mutation, 15 for ALK rearrangement, and 4 for ROS1 rearrangement (Table 3).

**Table 3:** Tyrosine-kinase inhibitor (TKI) therapy stratified for receptor tyrosine-kinase (RTK) mutation for the *n* = 49 patients with confirmed RTK mutation, TKI therapy, and stage IV cancer at data assessment

TKI drug	EGFR^a^ (*N* = 30)	ALK^b^ (*N* = 15)	ROS1^c^ (*N* = 4)
Afatinib	9	–	–
Gefitinib	6	–	–
Erlotinib	22	2	1
Crizotinib	0	13	4
Nintedanib	1	–	–
Buparlisib	–	–	–
Alectinib	–	1	–
ARQ197	1	–	–
Ceritinib	–	3	–
Osimertinib	2	–	–
PTK/ZK	–	1	–
Rociletinib	2	–	–
Carbozantinib	–	–	1
LDK378	–	1	–

^a^Epidermal growth factor receptor (EGRF) mutations

^b^Anaplastic lymphoma kinase gene (ALK) rearrangements

^c^Proto-oncogene receptor tyrosine-kinase (ROS1) rearrangements

Among the 30 individuals with EGFR mutation, therapy was conducted 9 times with *Afatinib*, 6 times with *Gefitinib*, 22 times with *Erlotinib*, and less often with other TKIs (e.g., *Nintedanib*, and *Osimertinib*). Among the 15 individuals with ALK rearrangement, therapy was conducted 2 times with *Erlotinib*, 13 times with *Crizotinib*, 3 times with *Ceritinib*, and less often with other TKIs (e.g., *Alectinib*). Among the four individuals with ROS1 rearrangement, therapy was conducted four times with *Crizotinib*, and one time with both *Erlotinib* and *Cabozantinib* (Tables 3, 4).

**Table 4:** Summary of tyrosine-kinase inhibitor (TKI) and therapy line-status of the *n* = 49 patients with confirmed receptor tyrosine-kinase (RTK) mutation, TKI therapy, and stage IV cancer at the time of the data assessment

TKI drug and therapy status		First line	Second line	Further lines	Last line	All lines
		(*N* = 49)	(*N* = 37)	(*N* = 13)	(*N* = 24)	
		*N*	*N*	*N*	*N*	*N*
Afatinib						
in Afatinib treatment		1	1	0	4	6
Afatinib treatment ended—no further treatment		1	0	0	0	1
Afatinib treatment ended—in further treatment		1	1	1	0	3
Gefitinib						
in Gefitinib treatment		1	0	0	2	3
Gefitinib treatment ended—no further treatment		0	0	0	0	0
Gefitinib treatment ended—in further treatment		3	0	0	0	3
Erlotinib						
in Erlotinib treatment		7	1	0	2	10
Erlotinib treatment ended—no further treatment		1	0	0	1	2
Erlotinib treatment ended—in further treatment		5	4	5	0	14
Crizotinib						
in Crizotinib treatment		1	5	0	6	12
Crizotinib treatment ended—no further treatment		0	1	0	1	2
Crizotinib treatment ended—in further treatment		1	3	0	0	4
Other TKI						
in other TKI treatment		0	4	0	4	8
other TKI treatment ended—no further treatment		0	0	0	0	0
other TKI treatment ended—in further treatment		1	3	1	0	5
All						
in TKI treatment		10	11	0	18	39
TKI treatment ended—no further treatment		2	1	0	1	5
TKI treatment ended—in further treatment		11	11	7	0	29
All lines		23	23	7	19	73
In clinical studies		5	5	3	2	15

### Quality of life and symptom burden

In group A, the mean score for global QOL was 57.1, while the mean score for physical functioning was 61.1. In the symptom scales, the mean score was 50.0 for fatigue, 33.3 for diarrhoea, 32.7 for pain, 31.7 for dyspnoea, 29.9 for appetite loss, 25.0 for rash, and 13.2 for nausea and vomiting.

Six individuals (12.2%) presented elevated symptoms of both anxiety and/or depression.

In group B, the mean score for global QOL was 68.8, while the mean score for physical functioning was 64.0. In the symptom scales, the mean score was 48.6 for fatigue, 35.6 for dyspnoea, 29.3 for pain, 20.7 for appetite loss, 11.8 for rash, 8.1 for nausea and vomiting, and 7.7 for diarrhoea.

23 individuals (19.0%) presented elevated symptoms of depression, and 18 (14.9%) of anxiety.

In group C, the mean score for global QOL was 57.5, while the mean score for physical functioning was 62.3. In the symptom scales, the mean score was 48.3 for fatigue, 43.2 for dyspnoea, 31.2 for pain, 18.5 for appetite loss, 13.7 for diarrhoea, 12.0 for rash, and 8.4 for nausea and vomiting. 104 individuals (21.0%) presented elevated symptoms of depression, and 90 (18.3%) of anxiety (Table 5).

**Table 5:** Mean scores and standard deviation (SD) for the considered scale in the European Organization for Research and Treatment of Cancer Quality of Life Questionnaire Core 30 (EORTC QLQ-C30) and symptom scale rash, as well as absolute and relative frequencies for Patient Health Questionnaire-4 (PHQ-4) considered for the analysis. Differences in mean scores and proportions, as well as 95% confidence intervals (CI) of the estimates, are reported for group comparison

Quality of life and symptom scales	Group A^a^		Group B^b^		Group C^c^		Difference in mean scores										
							Between groups A and B				Between groups A and C				Between groups B and C		
	Mean	SD	Mean	SD	Mean	SD	Estimate	95% CI			Estimate	95% CI			Estimate	95% CI	
								Lower	Upper			Lower	Upper			Lower	Upper
Global quality of life	57.1	21.4	68.8	22.8	57.5	24.6	− 11.7	− 19.19	− 4.21		− 0.4	− 7.56	6.76		11.3	6.47	16.13
Physical functioning	61.1	28.1	64.0	24.5	62.3	24.7	− 2.9	− 11.45	5.65		− 1.2	− 8.56	6.16		1.7	− 3.21	6.61
Fatigue	50.0	30.8	48.6	28.8	48.3	29.0	1.4	− 8.42	11.22		1.7	− 6.88	10.28		0.3	− 5.47	6.07
Nausea and vomiting	13.2	23.3	8.1	19.1	8.4	16.2	5.1	− 1.72	11.92		4.8	− 0.19	9.79		− 0.3	− 3.65	3.05
Pain	32.7	34.2	29.3	31.8	31.2	32.9	3.4	− 7.47	14.27		1.5	− 8.21	11.21		− 1.9	− 8.41	4.61
Appetite loss	29.9	37.8	20.7	30.4	18.5	28.3	9.2	− 1.73	20.13		11.4	2.79	20.01		2.2	− 3.52	7.92
Diarrhoea	33.3	37.0	7.7	17.2	13.7	25.7	25.6	17.39	33.80		19.6	11.31	27.89		− 6.0	− 11.07	− 0.93
Rash	25.0	32.6	11.8	27.9	12.0	26.7	13.2	2.92	23.48		13.0	4.59	21.41		− 0.2	− 5.82	5.42
Dyspnoea	31.7	26.1	35.6	26.1	43.2	27.2	− 3.9	− 13.05	5.25		− 11.5	− 19.86	− 3.14		− 7.6	− 13.23	− 1.97

PHQ-4	Group A^a^		Group B^b^		Group C^c^		Difference in proportions										
							Between groups A and B				Between groups A and C				Between groups B and C		
	*N*	%	*N*	%	*N*	%	Estimate	95% CI			Estimate	95% CI			Estimate	95% CI	
								Lower	Upper			Lower	Upper			Lower	Upper
Depression	6	12.2	23	19.0	104	21.0	− 6.8	− 18.33	4.73		− 8.8	− 18.64	1.04		− 2.0	− 9.86	5.86
Anxiety	6	12.2	18	14.9	90	18.3	− 2.7	− 13.85	8.45		− 6.1	− 15.88	3.68		− 3.4	− 10.60	3.80

^a^Group A: *N* = 49, patients with confirmed RTK mutation, TKI treatment, and stage IV cancer at data assessment

^b^Group B: *N* = 121, patients with no-RTK mutation, non-TKI treatment, and stage IV cancer at diagnosis or metastases

^c^Group C: *N* = 495, patients with no-RTK mutation, non-TKI treatment, and stage I-III cancer at diagnosis, no metastases

### Comparisons of quality of life between treatment groups

A medium relevant difference in mean score for global QOL in group A compared to group B was observed (− 11.7 [95% CI − 19.19, − 4.21]). Individuals in group A reported a little relevant higher symptom burden for appetite loss (+ 11.4 [95% CI 2.79, 20.01]) and a medium relevant lower symptom burden for dyspnoea (− 11.5 [95% CI − 19.86, − 3.14]) than individuals in group C. Individuals in group A presented medium relevant difference in mean score for diarrhoea (+ 25.6 [95% CI 17.39, 33.80] and + 19.6 [95% CI 11.31, 27.89], respectively) and moderate relevant difference in mean score for rash (+ 13.2 [95% CI 2.92, 23.48] and + 13.0 [95% CI 4.59, 21.41], respectively), when compared with individuals in groups B and C (Table 5).

## Discussion

This study aimed at exploring the QOL of a real-world population of lung cancer survivors with proven RTK genetic alterations and targeted TKI therapy, compared to lung cancer survivors with no-RTK alterations and no-TKI therapy.

Stage IV cancer survivors treated with TKI therapy reported clinically relevant lower global QOL than stage IV survivors treated with no-TKI therapy. This result disagrees with previous studies: Oizumi et al. (2012a) and Wu et al. (2018) observed a larger proportion of lung cancer patients treated with TKI improving their QOL over time, compared to patients treated with chemotherapy. These contrasting results might be explained considering the difference in the composition of the study populations. The aforementioned studies (Oizumi et al. 2012a; Wu et al. 2018) compared similar groups of patients, while in this study, survivors treated with TKI therapy differed both clinically and demographically from survivors with no-TKI therapy. According to clinical records, a larger proportion of survivors with TKI therapy (80%) was still in treatment at data assessment, compared to survivors with no-TKI therapy (27% and 9%, respectively). Treatment status has been previously associated with QOL of cancer survivors: Hechtner et al. (2019) observed a negative association between current/recent treatment and QOL in cancer survivors (*β* = − 7.9, *p* = 0.006). Therefore, the difference in proportion of survivors in treatment between the two groups might have negatively influenced the QOL among survivors with TKI therapy. Conversely, however, survivors with TKI therapy were also younger and more represented in higher income classes, compared to the survivors with no-TKI therapy. Age and income have been both associated with QOL in lung cancer patients and survivors (Hechtner et al. 2019; Pierzynski et al. 2018; ACTION study group 2017). Thus, these differences in the compared groups might have influenced—this time—positively the QOL among the survivors treated with TKI therapy.

Stage IV cancer survivors treated with TKI therapy presented a clinically relevant lower symptom burden for dyspnoea, than stage I–III survivors treated with non-TKI therapy. Similar results were reported in previous studies: Oizumi et al. (2012b) observed a longer time to deterioration for shortness of breath in patients treated with TKI compared to patients treated with carboplatin and paclitaxel. Wu et al. (2014, 2018) reported a longer time to deterioration and lower scores for dyspnoea in patients treated with TKI therapy compared to patients receiving chemotherapy. Yang et al. (2013) reported a significant improvement in dyspnoea in patients treated with TKI therapy compared to patients treated with chemotherapy. In this study, the significant difference was observed between stage IV survivors treated with TKI therapy and stage I–III survivors treated with chemotherapy. This might be explained considering that cancer stage has been found to have no predictive effect on the burden of dyspnoea (Smith et al. 2001). Conversely, the presence of respiratory comorbidities was associated with a higher burden of dyspnoea (*β* = 5.1, *p* = 0.008) (Hechtner et al. 2019). In this study, a smaller proportion of stage IV survivors treated with TKI (47%) reported having unspecified comorbidities, compared to stage I–III survivors treated with no-TKI (73%). Therefore, this difference between the compared groups should be considered when interpreting the lower burden of dyspnoea.

Survivors treated with TKI therapy presented a clinically relevant higher symptom burden for diarrhoea and rash compared to both groups of survivors treated with non-TKI therapy, and clinically relevant higher symptom burden for appetite loss compared to stage I–III cancer survivors treated with non-TKI therapy. These results are in accordance with what observed from previous works on the topic. Yang et al. (2013) observed patients treated with *Afatinib* reporting shorter time to deterioration for diarrhoea than patients treated with chemotherapy (HR 7.74, 95% CI 5.15, 11.63). Yan et al. (2015) observed patients with TKI therapy in addition to chemotherapy presenting a higher risk for rash than patients with only chemotherapy [risk ratio (RR) 7.43; 95% CI 4.56, 12.09]. In the interpretation of the results for the first two adverse effects, it must be again considered that a larger percentage of survivors treated with TKI were in treatment at the moment of the data assessment, compared to survivors with no-TKI therapy. Regarding the difference in appetite loss, it must be considered that the significant difference was present between two groups with different cancer stage at diagnosis.

Due to the explorative nature of this paper, no confounder analysis was considered. In addition, due to the limited number of stage IV cancer survivors treated with TKI, no stratified analysis (e.g. on treatment status) was conducted. Hence, the interpretation of the results of the comparisons between the groups must be conducted with caution, keeping in mind the composition of the study population.

The cross-sectional design was expected to better depict the real-world population of lung cancer survivors, in contraposition with most of the scientific literature on the topic based on clinical trials. This approach was considered of interest for clinicians, as it might provide different and integrative information to the clinical trial-based works, especially in terms of long-term effects of the therapy with TKIs on QOL and symptom burden, as well as the distribution of QOL and symptom burden over a heterogeneous population of cancer patients and survivors. However, this study design also limits the analysis. Besides the aforementioned limitations of the heterogeneity in the three groups, due to the specific focus on patients and survivors at least one year since the diagnosis, information regarding patients that succumbed to the disease within the first year was not retained. When interpreting the results, it is worth considering that the estimated effect estimates present always with large standard deviations and confidence intervals, respectively, indicating elevated statistical uncertainty. In this scenario, the clinical relevance of the mean score differences should be interpreted cautiously and alongside to the reported confidence intervals. In addition to this, due to the relatively recent development of TKI drugs compared to the time-frame of the data collection of the study, the group of patients with TKI therapy might be more recent than the other two groups. Finally, as we did not correct for multiple testing, there is an increased possibility of chance findings. For all these reasons, conclusions were drawn carefully.

## Conclusions

In our study, lung cancer stage IV survivors with proven RTK genetic alterations and targeted TKI therapy reported suffering more frequently from loss of appetite, diarrhoea and rash, and less frequently from dyspnoea, than lung cancer survivors treated with chemotherapy. In addition, lung cancer stage IV survivors with proven RTK genetic alterations and targeted TKI therapy reported a lower global QOL compared to lung cancer survivors treated with chemotherapy.

These results suggest that TKI therapy presents specific side effects which can impair the quality of life of lung cancer survivors. Therefore, an optimal management of these TKIs-specific side effects is to consider crucial, and not less important than the treatment of the side effects of chemotherapy. This is especially important in the context of upcoming use of TKI therapy in adjuvant settings, where relevance of QOL data is growing and optimized toxicity management is critical.

We specifically considered a cross-sectional design for this study, to allow the observation of a real-world lung cancer population. At the same time, the interpretation of results must be conducted with caution, considering the statistical uncertainty of the effect estimates and their clinical relevance, the heterogeneity of the study groups, as well as the absence of an analysis stratified by therapy and a confounder analysis.

## Data Availability

Not applicable.
